# Utilization of a Deoxynucleoside Diphosphate Substrate by HIV Reverse Transcriptase

**DOI:** 10.1371/journal.pone.0002074

**Published:** 2008-04-30

**Authors:** Scott J. Garforth, Michael A. Parniak, Vinayaka R. Prasad

**Affiliations:** 1 Department of Microbiology and Immunology, Albert Einstein College of Medicine, Bronx, New York, United States of America; 2 Department of Molecular Genetics and Biochemistry, University of Pittsburgh School of Medicine, Pittsburgh, Pennsylvania, United States of America; AIDS Research Center, Chinese Academy of Medical Sciences and Peking Union Medical College, China

## Abstract

**Background:**

Deoxynucleoside triphosphates (dNTPs) are the normal substrates for DNA synthesis catalyzed by polymerases such as HIV-1 reverse transcriptase (RT). However, substantial amounts of deoxynucleoside diphosphates (dNDPs) are also present in the cell. Use of dNDPs in HIV-1 DNA synthesis could have significant implications for the efficacy of nucleoside RT inhibitors such as AZT which are first line therapeutics for treatment of HIV infection. Our earlier work on HIV-1 reverse transcriptase (RT) suggested that the interaction between the γ-phosphate of the incoming dNTP and RT residue K65 in the active site is not essential for dNTP insertion, implying that this polymerase may be able to insert dNDPs in addition to dNTPs.

**Methodology/Principal Findings:**

We examined the ability of recombinant wild type (wt) and mutant RTs with substitutions at residue K65 to utilize a dNDP substrate in primer extension reactions. We found that wild type HIV-1 RT indeed catalyzes incorporation of dNDP substrates whereas RT with mutations of residue K65 were unable to catalyze this reaction. Wild type HIV-1 RT also catalyzed the reverse reaction, inorganic phosphate-dependent phosphorolysis. Nucleotide-mediated phosphorolytic removal of chain-terminating 3′-terminal nucleoside inhibitors such as AZT forms the basis for HIV-1 resistance to such drugs, and this removal is enhanced by thymidine analog mutations (TAMs). We found that both wt and TAM-containing RTs were able to catalyze Pi-mediated phosphorolysis of 3′-terminal AZT at physiological levels of Pi with an efficacy similar to that for ATP-dependent AZT-excision.

**Conclusions:**

We have identified two new catalytic functions of HIV-1 RT, the use of dNDPs as substrates for DNA synthesis, and the use of Pi as substrate for phosphorolytic removal of primer 3′-terminal nucleotides. The ability to insert dNDPs has been documented for only one other DNA polymerase, the RB69 DNA polymerase and the reverse reaction employing inorganic phosphate has not been documented for any DNA polymerase. Importantly, our results show that Pi-mediated phosphorolysis can contribute to AZT resistance and indicates that factors that influence HIV resistance to AZT are more complex than previously appreciated.

## Introduction

DNA polymerases are responsible for replicating and repairing an organism's genome (reviewed in [1]). Polymerases responsible for replication have properties that are different from those responsible for repair. Replicative polymerases are accurate and processive (multiple nucleotide incorporations) in their DNA synthesis, whereas repair polymerases are less accurate and distributive (insertion of single, or a few nucleotides). HIV-1, the causative agent of AIDS, uses a specialized DNA polymerase for its replication. This DNA polymerase is a reverse transcriptase, and is capable of synthesizing a DNA strand from either a DNA or RNA template. HIV-1 reverse transcriptase (RT) is a replicative DNA polymerase, but has a fidelity (the accuracy with which it copies the template strand) that is intermediate between typical replicative and repair polymerases [2], [3]. In common with all polymerases, the nucleotide to be added to the nascent DNA chain forms many interactions with the polymerase. These interactions include contacts between RT and portions of the incoming nucleotide distinct from the base [4], which stabilize the nucleotide in the active site without sequence specificity, although the interactions may influence polymerase fidelity. Recent results from our laboratory and others have demonstrated that one such interaction, between the K65 residue of RT and the γ-phosphate of the incoming nucleotide is not absolutely required for polymerase activity. Abolition of the interaction by deleting residues 65–69 in the fingers subdomain of the RT, or by point mutation of the K65 residue that interacts with the γ-phosphate, resulted in RT that retained activity, albeit much less than the wild type enzyme [5], [6]. The non-essential nature of this interaction suggests that the γ-phosphate of the incoming nucleotide may be dispensable, and therefore that a nucleoside diphosphate may be utilized as a substrate by RT.

Deoxynucleoside diphosphates are naturally present in the cell as an intermediate in the de novo synthesis of deoxynucleotide triphosphates [7]. The relative concentration of the nucleoside diphosphate compared to the triphosphate depends on the cellular localization; for example, more TDP than TTP is detected in the mitochondrial fraction, however, approximately 80% of cytosolic thymidine is present as TTP and 20% as TDP [8]. Utilization of a nucleoside diphosphate substrate by template-dependent polymerases is very unusual. To our knowledge, only one example of such a polymerase has been reported, phage RB69 DNA polymerase gp43 [9]. RB69 gp43 is a replicative DNA polymerase from polymerase family B, as distinct from the polymerase A family member, the *E. coli* DNA polymerase I, which is a repair polymerase [10].

**Figure 1:**
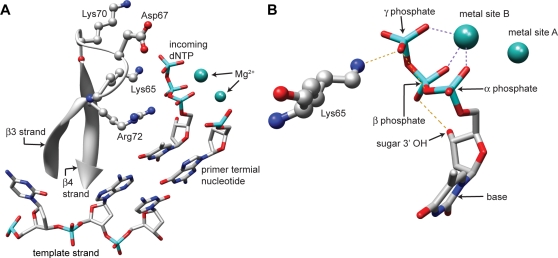
Interactions between the dNTP substrate and RT. A. Representation of the active site highlighting fingers subdomain residues involved in binding of the incoming nucleotide or AZT resistance. B. Detailed view of the interactions between the Lys65 residue, the incoming nucleotide and the catalytic metal ions. Metal ion coordination is depicted with a dashed purple line, hydrogen bonds with an orange line. Figures were generated from the published HIV-1 RT ternary structure (1rtd) [4] using the UCSF Chimera package [50].

Pyrophosphorolysis, the reverse reaction of polymerization, is the pyrophosphate-dependent excision of the 3′-terminal nucleotide from a DNA chain, and results in the release of this terminal nucleotide as a nucleoside triphosphate. The reverse reaction of polymerization utilizing dADP as a substrate would be inorganic phosphate-dependent phosphorolysis. Pyrophosphorolysis and nucleoside dependent phosphorolysis have a particular importance in the susceptibility of HIV-1 to nucleoside analog drugs, as RT mutations (thymidine analog mutations; TAMs) that increase the efficiency of these processes are responsible for the resistance of HIV-1 to thymidine analogs such as AZT [11], [12]. Clinically significant TAMs include combinations of M41L, D67N, K70R, T215F/Y, and K219Q. Wild-type HIV-1 RT can catalyze the removal of a chain-terminating dideoxynucleotide by either pyrophosphorolysis or nucleotide (ATP)-dependent phosphorolysis [11]. Introduction of TAMs increases RT-catalyzed ATP-dependent removal of chain-terminating NRTIs as well as reducing the inhibition of NRTI removal by the next complementary nucleotide [13]. Mutations of residues 67 and 70, both in the fingers subdomain and near residue K65 (Fig. 1A), were found to have the greatest effect on increasing unblocking [13], while a T215F substitution in the palm subdomain is largely responsible for the reduced inhibition by the next complementary nucleotide [14]. TAMs have a much smaller impact on the rate of pyrophosphorolysis than on the rate of ATP-mediated phosphorolysis [12], [13], [15].

In this report, we show for the first time that one of the most extensively studied DNA polymerases, HIV-1 RT, is in fact able to use deoxynucleoside diphosphates as a substrate for DNA synthesis. Importantly, we also show that RT catalyzes the reverse reaction, phosphorolytic removal of primer 3′-nucleotides mediated by inorganic phosphate (Pi). This latter observation has significant impact on the continued utility of nucleoside analog drugs as first line therapeutics for HIV-1 infection.

## Results

### HIV-1 RT can catalyze the insertion of deoxynucleoside diphosphates

**Figure 2:**
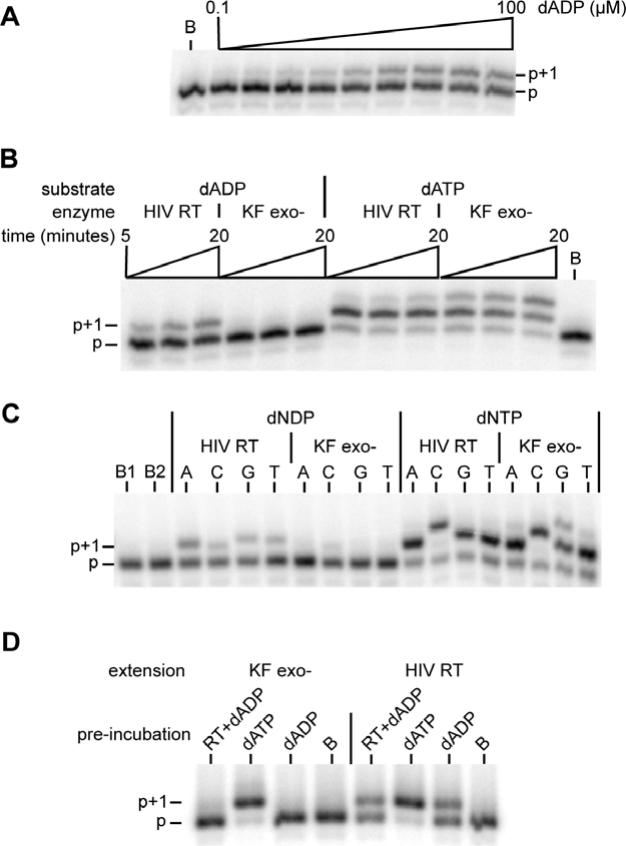
Utilization of a deoxynucleotide diphosphate substrate by HIV-1 RT. A. 5′ end-labeled primer, annealed to a template strand, was extended in the presence of a range of dADP concentrations by HIV-1 RT. The primer is extended by a single nucleotide, corresponding to the template ‘T’ at the +1 position. Extension reactions contained 10 nM wild-type enzyme, 5 nM primer-template, and were carried out for 10 minutes. Unextended primer is labeled ‘p’, the single nucleotide extension product is ‘p+1’. B. Single nucleotide extension assays of HIV-1 RT or KF exo- (50 nM each) with 10 µM of either dADP or dATP demonstrated no activity in the reaction containing KF exo- and dADP. Lane B, unextended primer. C. Single nucleotide extension assays utilizing dADP, dCDP, dGDP and dTDP incorporation opposite the complementary template nucleotide. Reactions were carried out with 100 µM of the appropriate dNDP or 0.2 µM dNTP, 50 nM enzyme and 5 nM template-primer. Lanes B1 and B2 contained RT and KF exo- respectively, with no nucleotide added. D. The RT preparation does not contain a contaminating nucleotide kinase activity. Nucleotides, which had been pre-incubated in the presence (labeled RT+ADP) or absence of HIV-1 RT, were used in a single nucleotide extension assay with either KF exo- or HIV-1 RT. Lane B: pre-incubation reaction contained no nucleotide. Unextended primer and primer extended by a single nucleotide are indicated ‘p’ and ‘p+1’ respectively.

In order to test the hypothesis that HIV-1 RT can utilize a nucleoside diphosphate substrate, we performed a single nucleotide extension assay with dADP on a DNA.DNA template primer. Extension of the 5′ radiolabeled primer by a single nucleotide was detected, and the reaction displayed typical Michaelis-Menten kinetics (*K*_m_ of 9.9±1.6 µM, *k*_cat_ 0.02±0.001 min^−1^, *k*_cat_/*K*_m_ 0.002 µM^−1^ min^−1^) (Fig. 2A). These values are to be compared with the kinetic constants that we previously published [5] for single nucleotide extension utilizing dATP and the same template-primer: *K*_m_ 0.02±0.004 µM, *k*_cat_ 2.3±0.15 min^−1^ and *k*_cat_/*K*_m_ 115 µM^−1^ min^−1^. As expected, no activity was observed with dAMP (result not shown), as has been demonstrated previously for HIV-1 RT [16]. The incorporated nucleotide could be further extended by RT in the presence of all dNTPs, and the reaction was template-dependent (data not shown). We believe that this is the first demonstration that HIV-1 RT can use a nucleoside diphosphate as a substrate for DNA synthesis.

The primer extension assays were performed using commercially available, HPLC purified, deoxyadenosine di- and triphosphates, further purified by ion exchange chromatography. However, the possibility that the dADP-dependent polymerase activity was due to dATP contamination in the dADP stock remained. To exclude this possibility, single nucleotide primer extension assays were performed with the 3′–5′ deficient derivative of Klenow fragment from DNA Pol I (KF exo-). KF exo- was used as a negative control because DNA Pol I has been demonstrated to be incapable of using a deoxynucleoside diphosphate substrate [17]. Under conditions in which activity was detected using RT, no extension was seen using KF exo-, demonstrating the absence of significant dATP contamination (Fig. 2B). Similar experiments were carried out utilizing FPLC purified dCDP, dGDP and dTDP (Fig. 2C) which showed that RT, but not Kf exo-, was capable of using each of the four dNDPs as substrates opposite the corresponding complementary template base. To exclude the possibility that the purified RT was contaminated with either a nucleotide or a kinase capable of phosphorylating the dADP to dATP, the dADP was treated by pre-incubation with the RT preparation, heat-treated to inactivate RT, and the material was used as a source of nucleotide in a primer extension assay by KF exo- (Fig. 2D, see RT+dADP lane). Again, no dADP-insertion activity was seen, demonstrating that the dADP utilization by RT was not due to a contamination in the enzyme preparation itself.

### Lysine residue in the fingers subdomain (K65) is essential for utilization of dNDPs

**Figure 3:**
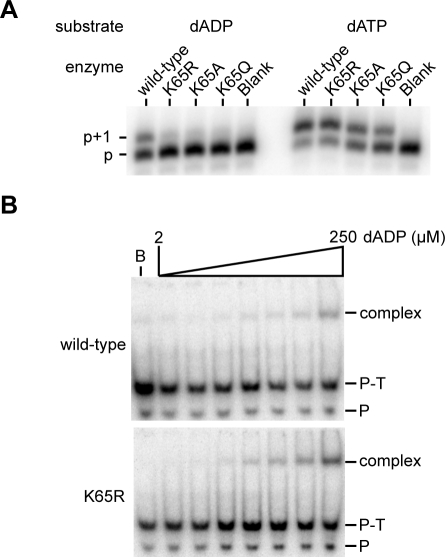
Mutations at the lysine 65 position abrogate dADP utilization. A. Single nucleotide extension assays were performed as in Figure 2A, but with enzyme concentrations of 10 nM and 2 nM for, respectively, dADP and dATP utilization. In both groups, the nucleotide concentration was 100 µM. B. Nucleotide binding measured by DEC formation. Reactions contained 20 nM wild-type or K65R mutant RT, and 2 to 250 µM dADP. After challenge with competitor DNA, reactions were separated by PAGE. Position of the complex is indicated. ‘P-T’ represents primer-template and ‘P’ is the unannealed primer. Reaction B contained no dADP.

We previously demonstrated that the K65 residue, which interacts with the γ-phosphate of the incoming dNTP (Fig. 1), is not essential for normal polymerization activity. In the context of an incoming nucleoside diphosphate, the K65 residue may be incapable of interacting with the shortened tail of a nucleoside diphosphate, and thus be entirely dispensable for activity. Therefore, we tested the nucleoside diphosphate insertion activities of K65 substitution mutant RTs in a single nucleotide extension assay. Three mutant RTs were tested including one with the conservative K65R substitution, and two non-conservative K65A and K65Q substitutions. No activity was detected with any of the mutants (Fig. 3A), suggesting that the lysine residue at position 65 is essential for the activity utilizing a nucleoside diphosphate. In order to assess whether the effect of the K65R substitution on dADP utilization is directly related to dADP-binding, dead-end complex (DEC) assays were performed (Fig. 3B). In the DEC assay, the formation of a stable complex between the enzyme and a primer-template in which the 3′ terminus is blocked is measured as a function of nucleotide concentration. Using this assay, the K65R substitution was demonstrated to have no gross effect on dADP binding, suggesting that this mutation did not have gross changes in the dNTP-binding pocket and that the observed lack of activity may be more directly related to the catalytic step. No binding could be detected with the K65A or K65Q mutant enzymes in the presence of dADP at 250 µM, the highest concentration used in our DEC assays for K65R mutant (result not shown). These results suggest that a basic residue at position 65 is necessary for dNDP-binding.

### HIV-1 RT catalyzes the inorganic phosphate-mediated phosphorolysis to release dNDP

**Figure 4:**
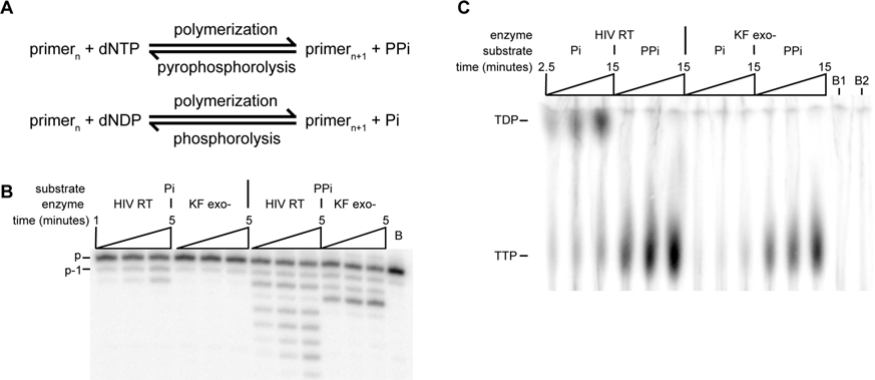
Pi-dependent phosphorolysis catalyzed by HIV-1 RT. A. Diagram showing that pyrophosphorolysis and Pi-dependent phosphorolysis are the reverse reaction of polymerization in which the substrate is a nucleotide triphosphate or diphosphate, respectively. B. Time course of phosphorolysis and pyrophosphorolysis reactions catalyzed by HIV-1 RT and KF exo-. Reactions were performed with 5mM PPi or K_2_HPO_4_, 10 nM enzyme and 5 nM template-primer. Aliquots of the reaction were stopped after 1, 2 and 5 minutes and analyzed by denaturing PAGE. Lane B contained a ‘no phosphate’ control in which template-primer was incubated with RT for 20 minutes. Full-length, 5′-end labeled primer is indicated ‘p’, phosphorolysis products shortened by a single nucleotide from the 3′ end are indicated ‘p-1’. C. Radiolabeled nucleotide products released from the primer 3′ terminus through a phosphorolysis reaction were separated by PEI-cellulose TLC. HIV-1 RT or KF exo- (5 nM) were incubated with primer template in the presence of 10 mM Pi or 150 µM PPi, and aliquots removed after 2.5, 5 and 15 minutes. Lanes B1 and B2 contained control reactions incubated for 15 minutes with RT and KF exo- respectively, in the absence of added phosphate. The position of TTP and TDP was determined by comparison to unlabeled standards.

Pyrophosphorolysis is the reverse of a polymerization reaction. While the forward reaction utilizes nucleoside triphosphate as the substrate, in the reverse reaction the newly formed primer (n+1) and pyrophosphate are used to regenerate dNTP and primer (n). The substrate for the reverse reaction of dADP utilization should be the inorganic phosphate and primer (n+1) to regenerate dADP and primer (n) (Fig. 4A). Therefore, we looked for inorganic phosphate-dependent (Pi-dependent) phosphorolysis of the 3′-terminal nucleotide from the primer DNA strand. Incubation of wild-type RT with template-primer, in the absence of deoxynucleotides, demonstrated the Pi-dependent removal of the terminal nucleotide from the radiolabeled primer (Fig. 4B). Significantly reduced Pi-dependent phosphorolysis was seen in reactions containing KF exo-, consistent with previous results that demonstrated an absence of Pi-dependent phosphorolysis in *E. coli* DNA Pol I [17]. In order to demonstrate that the Pi-dependent phosphorolysis was not due to a contamination of the reaction with pyrophosphate, the primer was 3′ labeled with [α-^32^P] TTP, and the nucleotide products of the phosphorolysis reaction analyzed by thin layer chromatography. If the Pi-dependent phosphorolysis observed was mediated by the contaminating PPi, the product released should be [α-^32^P] TTP, not [α-^32^P] TDP. The results clearly showed that the major product of Pi-dependent phosphorolysis was, as predicted, thymidine diphosphate (TDP), and that this product was not liberated in reactions containing Klenow fragment (Fig. 4C). Control reactions (lanes labeled PPi) showed that TTP was produced in pyrophosphorolysis reactions by both HIV-1 RT and KF exo-.

**Figure 5:**
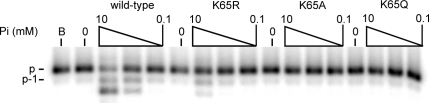
Influence of K65 substitutions on Pi-dependent phosphorolysis. A. Pi-dependent phosphorolysis is inhibited by substitutions of K65. 5′-end labeled primer was incubated with RT (10 nM) and a range of inorganic phosphate concentrations. Lane ‘B’ contained no enzyme, lane 0 contained no Pi.

Reverse transcriptase containing the conservative K65R mutation was capable of limited phosphorolysis, however the nonconservative K65A and K65Q mutations abolished the activity (Fig. 5). Thus, the ability of these mutants to insert dADP correlates with their abilities to catalyze the reverse reaction–Pi-dependent phosphorolysis.

### Inorganic phosphate can be a donor for AZT-excision by HIV-1 RT

**Figure 6:**
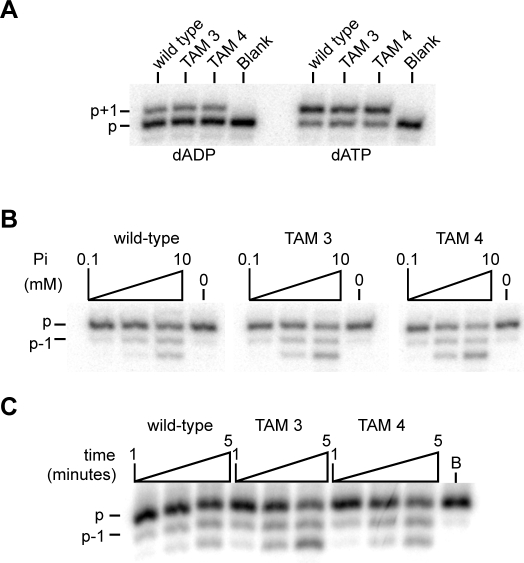
TAM mutants utilize dADP and perform Pi-dependent phosphorolysis. A. Single nucleotide insertion assays performed with 100 µM nucleotide and 10 nM or 2 nM each enzyme, for dADP and dATP reactions respectively. ‘p’ indicates unextended primer and ‘p+1’, the single nucleotide extension product. B. Pi-dependent phosphorolysis reactions contained 10 nM each enzyme, and 0, 0.1, 1 or 10 mM Pi. Full length 5′ end labeled primer is indicated ‘p’, primer with a single nucleotide excised from the 3′ end is shown as ‘p-1’. C. Time course reactions for the Pi-dependent phosphorolysis contained 10 nM each enzyme and 10 mM Pi.

**Figure 7:**
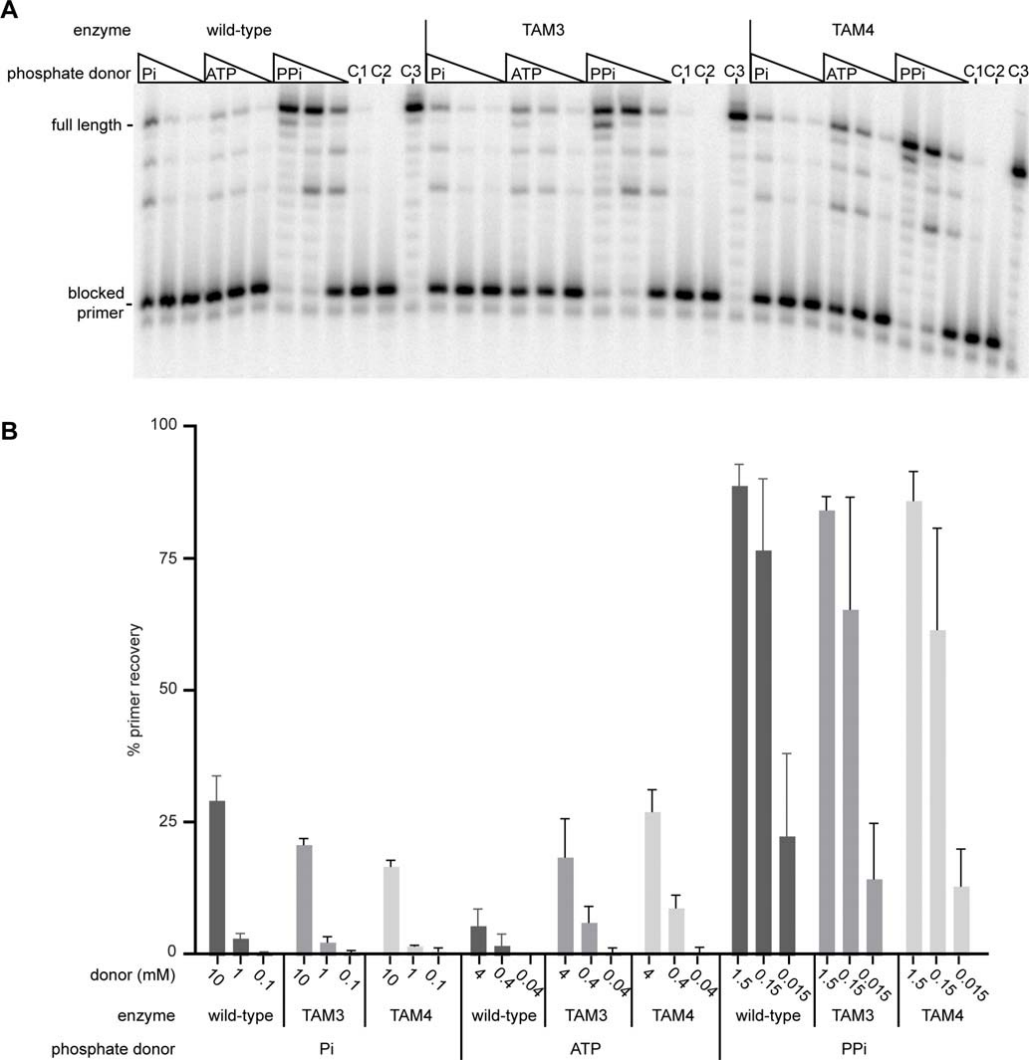
Pi-dependent primer unblocking by wild type and TAM mutants. 5′ end labeled primer was blocked with AZT, followed by incubation with a phosphate donor (Pi, ATP or PPi) and dNTPs. Unblocked primer is extended to the end of the template, resulting in the ‘full length’ product. Control lanes C1, C2 and C3 contained no phosphate donor, no dNTPs and no AZT respectively. B. Graphical representation of the results from three independent primer unblocking experiments. Error bars show the standard deviation of the results.

HIV resistance to AZT is commonly associated with a cluster of mutations in the reverse transcriptase, known as TAMs (thymidine analog mutations). These mutant RTs lead to resistance by increasing the efficiency of excision of the chain-terminating AZT monophosphate from the terminus of the nascent DNA chain by ATP-mediated phosphorolysis. The TAM3 (M41L/L210W/T215Y) and TAM4 (D67N/K70R/T215F/K219Q) enzymes were capable of using a dADP substrate (Fig. 6A) and efficiently performed the Pi-dependent phosphorolysis reaction (Fig. 6B and 6C). These enzymes were assayed for primer rescue activity, a test of their ability to remove a chain-terminating inhibitor, using inorganic phosphate as the donor. As shown in Figure 7, the wild-type and both TAM reverse transcriptases were capable of Pi-dependent primer rescue. In the wild-type enzyme, the Pi-dependent recovery was more efficient than ATP-dependent phosphorolysis, but far less efficient than pyrophosphorolysis. As expected, the TAM RTs showed increased utilization of ATP, compared to wild-type use of ATP as a donor in the primer rescue experiment. However, the TAM enzymes were less efficient than the wild type enzyme in Pi-dependent rescue (Fig. 7B). Nevertheless, Pi dependent activity contributed significantly to primer rescue by the TAM enzymes.

## Discussion

Our results demonstrate that HIV-1 reverse transcriptase can utilize a deoxynucleoside diphosphate substrate in the polymerization reaction. Although the reaction is less efficient than polymerization using a deoxynucleoside triphosphate substrate, we demonstrate that the activity is an inherent property of the RT, and is not due to either the dATP contamination of the dADP, or a kinase contamination of the RT preparation. Evidence that the dADP preparation was not contaminated with dATP included FPLC analysis and the absence of this activity in KF exo- reactions with dADP. We have shown that incorporation by HIV-1 RT is detectable with a dADP concentration of 100 nM (Fig. 2A); no activity is seen with Kf exo- even at 10 µM dADP (Fig. 1B). Further, we demonstrated that the RT also performs Pi-dependent phosphorolysis and Pi-dependent excision of AZT.

Our data are surprising in that there is considerable evidence that the γ-phosphate of a dNTP is critical for nucleotide binding to RT. Substantial interactions involving the dNTP γ-phosphate, the RT bound catalytic metal ion, and residues K65 and D113 of RT were suggested by the crystal structure of RT-template/primer-dNTP ternary complex [4]. Interactions between the γ-phosphate of the incoming nucleotide and the polymerase active site have been shown to be crucial for stable nucleotide binding in both human DNA polymerase α and the *E. coli* Klenow fragment; much increased binding of nucleotide triphosphates was observed compared to diphosphates or monophosphates [18], [19]. Interestingly, this difference in affinity was only observed for complementary nucleotide addition, suggesting that addition of the correct nucleotide induced a conformational change in the protein, and that the γ-phosphate plays a crucial role in this conformational change. Further evidence for the role of the dNTP γ-phosphate in binding and positioning of the dNTP for DNA synthesis has been provided by mutation of the K65 residue to abolish the interaction [5] and by modification of the nucleotide γ-phosphate [20]. In both cases, increased fidelity resulted from reducing or abolishing the interaction between RT and the γ-phosphate. In the absence of the non-informational interaction between RT and the γ-phosphate, the nucleotide binding is hypothesized to be dependent on other, sequence-specific interactions between the incoming nucleotide and the enzyme [5].

Previous reports of a DNA polymerase utilizing a deoxynucleoside diphosphate as a substrate in a template dependent manner are sparse. Kornberg's group reported the unprimed DNA synthesis of an alternating dAT polymer, which was more efficient with dADP than dATP [21]. Subsequently, a dADP transferase activity was reported in some preparations of *E. coli* DNA polymerase I, which synthesized poly dA-dT without requiring a template or primer, apparently using dADP as the substrate [22]. More recently, it has been reported that the phage RB69 DNA polymerase is capable of using a dNDP substrate, although the reaction times were considerably longer than those reported here for HIV-1 RT [9], [23]. The crystal structure of this DNA polymerase revealed that the phosphate groups of the incoming nucleotide were stabilized in a similar manner to that found in HIV-1 reverse transcriptase; the phosphates are sandwiched between two divalent cations and three basic side-chains [24]. However, there is a significant difference in interactions between the phosphate groups and the enzyme in the course of nucleotide binding by RT, compared to the above polymerases; while RT stabilizes nucleotide binding by interactions involving the β3–β4 loop, the corresponding structure in polymerases from family A and B is an α helix [10].

Utilization of dADP as a substrate by HIV-1 RT is clearly much less efficient than its use of dATP; there are numerous probable factors involved in this reduced activity. First, the nucleoside diphosphate is bound with a much lower efficiency than the triphosphate nucleoside. In a ternary complex formation assay, the apparent dissociation constant (*K*_d,app_) for dADP was greater than 100 µM (Fig. 3B); by contrast, a similar experiment in which binding of dATP was measured demonstrated a *K*_d,app_ of approximately 9.2 µM [5]. Second, one of the two metal ion binding sites in the polymerase domain of HIV-1 RT (site B) is filled by a metal ion coordinated by the α, β and γ phosphates of the incoming nucleotide [4], [25], [26]. However, in the case of a nucleoside diphosphate, this coordination by the α and β phosphates of the nucleotide may lead to a positioning that is initially incorrect for catalysis, and rearrangement of metal ion-RT residue coordination may be needed for the reaction to proceed. Precedence for such a rearrangement comes from *E. coli* creatine kinase in which the metal ion rearranges from a β,γ to α,β coordination with the bound nucleotide [27]). An additional affect on the catalysis step may be due to alteration in the geometry of the β phosphate in the active site, affecting the formation of a catalytically important, but nonessential, hydrogen bond between a non-bridging oxygen on the β phosphate and the 3'OH of the incoming nucleotide (Fig. 1B) [28]. Finally, the metal ion in polymerase site B, coordinated with the β and γ phosphates, is proposed to enable the expulsion of the pyrophosphate leaving group [29]. This could be less efficient when the substrate is dADP, and the leaving group is Pi.

When DPO4, a polymerase from the Pol Y family, was crystallized with ddADP in the active site, the α-phosphate of the nucleotide was in the position that would normally be occupied by the γ-phosphate of a nucleotide triphosphate [30]. This arrangement would prevent nucleotidyl transfer, and so cannot be the case with dADP in the wild-type HIV-1 RT active site. Instead, it would appear that to some extent the α and β-phosphates of the dADP are coordinated similarly to their position when dATP is the substrate, as polymerization can occur. In polymerase β it has been suggested that triphosphate-sugar binding occurs before there is a check for complementarity in nucleotide insertion [31]. Subsequently, it has been demonstrated that the tridentate coordination of the nucleotide phosphates by the nucleotide-binding metal ion occurs in an ordered manner; the initial electrostatic interaction is with the γ-phosphate, followed by the β and α in turn. Our data suggests that this order is not necessarily rigid and that the γ-phosphate of the incoming nucleotide is not absolutely required for the nucleotidyl transfer to take place.

Substitutions of K65, the residue that ordinarily interacts with the γ-phosphate of the incoming nucleotide, have a dramatic affect on dADP utilization (Fig. 3A). We initially hypothesized that the K65 residue is essential because the lysine residue is required to interact with the β phosphate of the incoming dNDP, and that the K65R substitution abolishes the interaction, thus the deoxynucleoside diphosphate cannot be bound in the active site. However, using the dead end complex assay we demonstrated that the K65R mutation has no obvious effect on dADP binding (Fig. 3B), suggesting that the substitution instead directly affects catalysis. With a nucleoside triphosphate substrate, Selmi et al. demonstrated that the conservative K65R mutation has no affect on nucleotide binding, but it has a small affect on catalytic efficiency through reduction of the catalytic rate constant *k*_pol_. This reduction in efficiency is vastly increased, compared to the wild-type, with a dideoxynucleotide substrate. In the dideoxynucleotide, there is no hydrogen bond between the sugar and the non-bridging oxygen on the β phosphate of the incoming nucleotide (due to the absence of the sugar 3′ OH) and, as discussed above, absence of this interaction has a significant influence on catalysis [28]. Structural evidence (Sarafianos, S.G., personal communication) supports the specific affect of the K65R substitution on catalysis; in the absence of the stabilizing interaction between the 3'OH and the β phosphate of the nucleotide, R65 interacts instead with the α phosphate and the R72 residue, directly affecting catalysis. We speculate that this affect on *k*_pol_ may also be seen when dADP is the substrate, if the 3'OH-β phosphate stabilizing bond is no longer formed with this nucleotide.

The reverse reaction of polymerization utilizing a dNTP substrate is pyrophosphorolysis. In an analogous manner, Pi-dependent phosphorolysis is the reverse reaction of dADP utilization. We have shown that RT is capable of performing Pi-dependent phosphorolysis, and confirmed that the released product is, as expected, a nucleoside diphosphate (Fig. 4). As far as we are aware, this is the first demonstration of Pi-dependent phosphorolysis by a DNA polymerase. The most similar reported activities are pyrophosphorolysis by HIV-1 RT using either hypophosphoric acid [32] or phosphonoacetic acid [33] as a substrate. DNA-dependent RNA polymerase from *E. coli* has also been shown to catalyze the release of a nucleoside monophosphate from a DNA strand when the phosphate donor is a pyrophosphate analog containing an arsono group [34]. Additionally, as the RB69 DNA polymerase can utilize a nucleoside diphosphate substrate for polymerization [9], [23], it is probable that it can catalyze Pi-dependent phosphorolysis, but to our knowledge this has not been demonstrated. We observed a reduction in Pi-dependent phosphorolysis in the K65R mutant RT, and no observable phosphorolysis in the non-conservative substitutions. Although the K65R mutation is associated with AZT resistance in vitro [35], the mechanism for this resistance is through reduced efficiency of incorporation of the analog. The K65R mutation has been demonstrated to decrease pyrophosphorolysis [6] and ATP-dependent primer rescue [36], and re-sensitizes viruses carrying the TAM mutations to AZT [37], [38].

The concentration of inorganic phosphate (Pi) required to observe Pi-dependent phosphorolysis in vitro (1 to 10 mM) is within the range expected intracellularly. The intracellular Pi concentration varies according to both cell type and extracellular phosphate concentration, within a range of 0.7 to 3 mM [39], [40]. The intracellular pyrophosphate (PPi) concentration is typically stated to be between 120 and 150 µM in human lymphocytes [41]. However, recent data has shown that the PPi concentration is actually much lower; between 8 to 12 µM in unstimulated T cells, and between 55 to 79 µM in highly stimulated T cells [42]. RT-catalyzed phosphorolysis and primer rescue are observed in vitro even using these lower concentrations of PPi. Furthermore, recent work has shown that HIV-infected patients suffer from severe biochemical abnormalities, including hypophosphatemia [43]. The possible influence of these biochemical abnormalities on intracellular concentration of phosphate and phosphate-containing molecules is unknown.

We have also demonstrated that HIV-1 RT can mediate Pi-dependent primer rescue, and that the wild-type enzyme utilizes inorganic phosphate and ATP to a similar extent in this reaction. However, it is clear that the increase in chain-terminating nucleoside excision by the TAM mutants when using ATP as a substrate is not observed when using Pi as a substrate. It is possible that other TAM mutations (including those not tested here, as well as those that are as yet undiscovered) in the reverse transcriptase could enhance Pi-dependent primer rescue, and thus have important consequences in vivo. This would be analogous to the enhancement in efficiency in utilization of ATP as a substrate for AZT resistance by the TAM mutants compared to the wild-type enzyme. Obviously, mutations in HIV-1 RT that enhance nucleotide excision via Pi-dependent phosphorolysis have not been identified to date.

Meyer et al. demonstrated that many different nucleotides, including deoxynucleotides and nucleoside diphosphates, have the potential to be used by HIV-1-RT as a phosphate donor for the excision of AZT [11]. RT has been previously shown to use either PPi or ATP as a phosphate donor for excision of AZT from a chain terminated primer in vitro at physiologically relevant concentrations [42]. Circumstantial evidence suggests that ATP is the in vivo donor, as only excision using ATP as the donor is enhanced in the TAM RTs [13], and the TAM mutants are associated with AZT resistance in cell culture-based HIV replication assays. However, the nucleotide resulting from PPi dependent excision of AZT is AZT-triphosphate, and from ATP-dependent excision is a dinucleoside tetraphosphate (AZTp4A). AZT-triphosphate is obviously a substrate of the RT, and dinucleoside tetraphosphates have also been shown to be substrates for DNA chain elongation by RT [44]. More recently the nucleotide product of AZT excision, AZTp4A, was also specifically shown to be an efficient substrate for RT [45]. If a variant RT were to utilize inorganic phosphate as the phosphate donor in AZT excision, the released nucleotide would be AZT-diphosphate. The data we have presented in this paper show that RT can use a nucleoside diphosphate as a substrate for elongation, but with a much lower efficiency than a nucleoside triphosphate. This raises the possibility that a Pi-dependent excision mechanism could be an efficient mechanism for the removal of chain-terminating nucleotide analogs by HIV-1 RT.

## Materials and Methods

*Nucleotides, oligonucleotides and commercial enzymes* dADP, dCDP, dGDP, dTDP (dNDPs) and dAMP were purchased from MP Biomedicals. AZTTP was purchased from Moravek Biochemicals. In experiments comparing polymerase activity with dAMP, dNDP and dATP, the nucleotides were further FPLC purified as described below. Primers (Invitrogen and IDTDNA) were gel purified. Nucleoside triphosphates were purchased from CLP (Mercury Ultra-Pure nucleotides). Exonuclease negative Klenow fragment (KF exo-) was purchased from NEB. ATP and sodium pyrophosphate were purchased from Sigma-Aldrich. Inorganic pyrophosphatase was purchased from Roche.

### Protein purification

All proteins, except the TAM mutants, were purified as described in Garforth et al.[5]. The construction of expression plasmids and the purification of TAM mutant RTs was previously described [12], [46].

### Purification of dAMP, dNDPs and dATP

Purification of the nucleotides was carried out using an Äkta Explorer (Amersham Biosciences) at room temperature. A 1.25 mM solution of each nucleotide (250 µl) was applied to a Mono Q HR 5/5 column in 10 mM Tris, pH 8.0. The nucleotides were eluted from the column with a linear gradient from 0 to 200 mM NaCl in 40 column volumes. Peak elution of the nucleotides was at a NaCl concentration of 60 mM, 110 mM and 130 mM for dAMP, dADP and dATP respectively. The peak fractions, approximately 2 ml total for each nucleotide, were combined, quantified by spectrophotometry and used in the single nucleotide insertion assay.

### Single nucleotide insertion assays

Polymerase assays measuring the extension of a primer by a single nucleotide were carried out essentially as described [5]. Briefly, a 5′ ^32^P end-labeled primer (5′ ACGCCAGGGTTTTCCCAGTCACGACGTTGTAAA 3′) was annealed to the oligonucleotide Template1 (5′ CCCG**X**TTTACAACGTCGTGACTGGGAAAACCCTGGCGT 3′) at a 1:1.5 molar ratio. The residue indicated with ‘X’ (in bold) in the sequence was complementary to the incoming nucleotide. Different oligonucleotides with each of the four nucleotides at this position were employed. Enzyme (10 nM) and template-primer (5 nM) were incubated together in reaction buffer A (50 mM Tris-HCl (pH 8.0), 80 mM KCl, 6 mM MgCl_2_, 1 mM DTT, 0.1 mg/ml ultra-pure BSA (Ambion)) and a single nucleoside (either dATP or dADP, concentration specified in the figure legends). The reaction products were separated on a denaturing 10% acrylamide sequencing gel, and exposed to a PhosphorImager screen. In single nucleotide insertion assays using pre-treated nucleotides, the nucleotide added to the primer extension reaction was first incubated at 37°C for 15 minutes in reaction buffer A containing 250 µM dADP or 10 µM dATP, and RT (as required) at a concentration of 10 nM. The pre-incubation reactions were heat treated at 90°C for 3 minutes, and then added to extension assays such that the final nucleotide concentration was 50 µM (dADP) or 2 µM (dATP). The extension assays were performed in reaction buffer A, and contained 20 nM of HIV-1 RT or KF exo- incubated at 37°C for 15 minutes.

### Dead End Complex (DEC) formation

Reactions were performed essentially as described previously [5]. Briefly, primer (identical sequence as above) was 5′ end labeled with ^32^P, and blocked at the 3′ end with ddADP. The primer was annealed to Template2 (5′ CGCAGTATCCCGTTTTACAACGTCGTGACTGGGAAAACCCTGGCGT). 20 nM enzyme was incubated at 37°C for 5 minutes with 0.1 nM template primer in buffer A, DEC formation was initiated by the addition of dADP and the reaction incubated for a further 10 minutes. Finally a half volume of competitive trap mixture (12 µg/ml poly rA and 3 µg/ml oligo(dT) in 10 mM Tris, pH 8.0, 100 mM KCl, 30% glycerol and 50 µg/ml bromophenol blue) was added, the reactions were incubated for 5 minutes at 37°C, and then placed on ice. Reactions were analyzed by 6% native PAGE in 0.5x TBE, and visualized with a PhosphorImager.

### Phosphorolysis

Inorganic phosphate- and pyrophosphate-dependent phosphorolysis were analyzed by measuring the removal of the 3′ terminal nucleotide from a 5′ end-labeled primer, and separating the reaction products by 10% denaturing PAGE. Reactions were essentially as described for single nucleotide extension assays, but with no added nucleotide. Inorganic phosphate (K_2_HPO_4_) or sodium pyrophosphate was added to each reaction as described in the Figure legends. In order to analyze the phosphorolysis reactions by TLC, primer-Template1 described above was first annealed at a molar ratio of 1∶2, and labeled in a reaction containing 20 pmol primer, 40 pmol template, 60 mM Tris pH 8.0, 50 mM KCl, 6 mM MgCl_2_, 1 mM DTT, 0.1 mg/ml BSA, 120 µCi α^32^P TTP (3000 Ci/mmol, 10 mCi/ml) and 5 units KF exo- in a volume of 40 µl. The reaction was incubated at 37°C for 30 minutes, then heat inactivated at 80°C for 10 minutes. The KCl concentration was increased to 100 mM, and the primer-template annealed by slow cooling to room temperature. The annealed primer-template was separated from unincorporated radiolabeled TTP using a Micro Bio-Spin 30 column (Bio-Rad), which was pre-equilibrated with 20 mM Tris, pH 8.0, 100 mM KCl. The primer-template concentration was measured by scintillation counting, and used at a concentration of 5 nM in phosphorolysis reactions containing 10 nM wild type HIV-1 RT or KF exo-. With the exception of the primer-template, reaction components were identical to the PAGE-analyzed reactions. Reactions were initiated by the addition of K_2_HPO_4_ to a concentration of 10 mM, or 150 µM PPi, incubated at 37°C, and aliquots were removed at time intervals and stopped by the addition of EDTA to 23.3 mM. 2.5 µl of each stopped aliquot was applied to a fluorescent cellulose-PEI TLC plate (Selecto Scientific) and developed for 2.5 hours in 0.2 M K_2_HPO_4_, 0.35 M boric acid, 50 mM EDTA (modified from [47]). The samples were visualized by exposure to a PhosphorImager screen, and identified by comparison to unlabelled TDP and TTP standards, which were visualized by fluorescence.

### Primer rescue

Deoxynucleotides, AZTTP and ATP were treated with inorganic pyrophosphatase (Roche) essentially as described [48], except that pyrophosphatase was removed by filtration through a Vivaspin 500 10k MWCO ultrafiltration unit (Vivascience). Primer rescue experiments were performed using a protocol similar to that in Mas et al. [49]. Five nanomolar primer-template (primer as described above, template sequence 5′ CGCAGTATCCCGATTTACAACGTCGTGACTGGGAAAACCCTGGCGT) was blocked by incubation (10 minutes, 37°C) with 10 nM HIV-1 RT (wild-type or mutant, as specified) and 5 µM AZTTP in reaction buffer B (reaction buffer B was similar to buffer A, but contained 32 mM KCl). Primer recovery was initiated by simultaneous addition of 10 µM dNTPs and PPi, K_2_HPO_4_ or ATP as specified, and extended for 10 minutes at 37°C. Reactions were stopped by addition to 1.5 volumes 95% formamide loading buffer, and separated by denaturing 10% PAGE. The reaction products were visualized by exposure to a PhosphorImager screen.
